# 

**Published:** 1886-04

**Authors:** 


Vol. XL. APRIL, 1886. No. 4.

### THE DENTAL REGISTER

A MONTHLY JOURNAL,

DEVOTED TO THE INTERESTS OF THE PROFESSION.

EDITED BY

J. TAFT, D.D.S.

PUBLISHED BY
SAM'L A. CROCKER & CO.,
Successors to Spencer & Crocker,
OHIO DENTAL AND SURGICAL DEPOT,
Nos. 117, 119, 121 WEST FIFTH STREET, CINCINNATI, OHIO.

TERMS:—$2.00 per Annum, in Advance. Single Copies, 25 cts.

